# A Prospective Study on the Incidence of Postoperative Venous Thromboembolism in Korean Gastric Cancer Patients: An Inquiry into the Application of Western Guidelines to Asian Cancer Patients

**DOI:** 10.1371/journal.pone.0061968

**Published:** 2013-04-17

**Authors:** Jin Won Kim, Eun Ju Chun, Sang Il Choi, Do Joong Park, Hyung-Ho Kim, Soo-Mee Bang, Min Jeong Kim, Ju-Hee Lee, Moon-Soo Lee, Jeong-Ok Lee, Yu Jung Kim, Jee Hyun Kim, Jong Seok Lee, Keun-Wook Lee

**Affiliations:** 1 Department of Internal Medicine, Seoul National University Bundang Hospital, Seoul National University College of Medicine, Seoul, Republic of Korea; 2 Department of Radiology, Seoul National University Bundang Hospital, Seoul National University College of Medicine, Seoul, Republic of Korea; 3 Department of Surgery, Seoul National University Bundang Hospital, Seoul National University College of Medicine, Seoul, Republic of Korea; 4 Department of Surgery, Eulji University Hospital, Daejeon, Republic of Korea; University of Texas MD Anderson Cancer Center, United States of America

## Abstract

Several Western guidelines recommend the routine use of pharmacologic thromboprophylaxis for cancer surgery patients to prevent venous thromboembolism (VTE). However, the necessity of routine pharmacologic perioperative thromboprophylaxis in Asian gastric cancer (GC) patients has not been clearly determined. To determine the necessity of routine perioperative pharmacologic thromboprophylaxis in Korean gastric cancer patients, the incidence of postoperative VTE was prospectively evaluated in gastric cancer patients receiving surgery. Among 610 GC patients who had received surgery, 375 patents underwent routine duplex Doppler ultrasonography (DUS) on days 5–12 following surgery to detect VTE and then VTE-related symptoms and signs were checked at 4 weeks after surgery (cohort A). The 235 patients that declined DUS were registered to cohort B and the occurrence of postoperative VTE was retrospectively analyzed. In cohort A, symptomatic or asymptomatic VTE until 4 weeks after surgery was detected in 9 patients [2.4%; 95% confidence interval (CI); 0.9–3.9]. Tumor stage was a significant factor related to VTE development [stage I, 1.4%; stage II/III, 2.4%; stage IV, 9.7% (P = 0.008)]. In multivariate analysis, patients with stage IV had a higher postoperative VTE development [odds ratio, 8.18 (95% CI, 1.54–43.42)] than those with stage I. In cohort B, a low incidence of postoperative VTE was reaffirmed; only one postoperative VTE case (0.4%) was observed. In conclusion, the incidence of postoperative VTE in Korean GC patients was only 2.4%. Risk-stratified applications of perioperative pharmacologic thromboprophylaxis are thought to be more appropriate than the routine pharmacologic thromboprophylaxis in Korean GC patients receiving surgery.

## Introduction

Venous thromboembolism (VTE), including extremity deep vein thrombosis (DVT) and pulmonary embolism (PE), is attributed to several risk factors including old age, immobilization, surgery and others [1]. Especially in cancer surgery, the risk for VTE increases during the perioperative period [2], [3]. Therefore, several Western guidelines recommend the routine use of pharmacologic thromboprophylaxis for cancer surgery patients to prevent VTE [3], [4], [5], [6], [7].

Gastric cancer (GC) is particularly prevalent in eastern Asia. According to Western guidelines, all GC patients should receive pharmacologic prophylaxis such as low molecular weight heparin (LMWH) [3], [4], [5], [6]. Although there is no firm evidence from prospective studies, many Asian cancer surgeons believe that the incidence of postoperative VTE is not so high as they must follow Western guidelines. In our previous retrospective study, postoperative VTE was observed in only 0.2% of GC patients receiving surgery [8]. Our data strongly suggested that the incidence of postoperative VTE in Korean GC patients is much lower than that of Western patients [8], in whom the incidence has been reported to be a lot higher [9], [10]. Since most previous studies insisting on a low incidence of VTE in Asian patients have been retrospectively conducted [8], [11], the necessity of routine pharmacologic perioperative thromboprophylaxis in Asian cancer patients has not been clearly determined.

Due to ethnic differences in incidences of VTE between Asian and Western cancer patients, studies focusing on VTE in Asian patients are clearly required. Moreover, as GC is prevalent in Asia, large prospective studies on the incidence of postoperative VTE in Asian GC patients are required to justify risk-stratified application of perioperative pharmacologic thromboprophylaxis.

## Materials and Methods

### Patient population; a prospective cohort (cohort A)

This prospective study, conducted at Seoul National University Bundang Hospital (SNUBH), was carried out to investigate the postoperative incidence of VTE in GC patients. The pharmacologic prophylaxis for VTE in GC patients receiving surgery was not routine clinical practice at SNUBH. Patients who were admitted for GC surgery and met the eligibility criteria were consecutively enrolled between May 2010 and July 2011.

Patients with ≥ 20 years-of-age and who had pathologically confirmed adenocarcinoma of the stomach or gastroesophogeal junction were included. All patients received major abdominal cancer surgery for curative or palliative intent. Major surgery was defined as a surgical procedure lasting > 30 minutes [3]. All patients did not receive prophylactic pharmacologic anticoagulation. However, mechanical prophylaxis (elastic bandage or stockings) were allowed. The exclusion criteria were as follows: history of VTE, a known hypercoagulable state or congenital thrombophilia; concurrent VTE at the time of admission for GC surgery; a prior or concomitant malignancy, except for patients who were disease-free for 5 years after curative therapy; a history of taking antiplatelet or anticoagulant agents less than 2 days prior to the operation; comorbidities that required pharmacologic anticoagulation during the perioperative period (i.e., atrial fibrillation or cerebrovascular infarct); and pregnancy.

In the prospective cohort (cohort A; N = 375), all patients underwent duplex and color Doppler ultrasonography (DUS) on lower extremities to screen for DVT regardless of postoperative symptom development. All patient demographics and laboratory data were collected before surgery. A variation of the Elixhauser Comorbidity index was used for comorbidities [12], [13], [14]. Comorbidities that indicated the presence of another cancer or transient conditions (i.e., electrolyte disturbance or transient arrhythmia) were excluded; however, hyperlipidemia was included as one comorbidity entity [11]. The tumor stage was based on the final pathology reports.

### The detection of VTE in the cohort A

All cohort A patients underwent a DUS between 5–12 days following GC surgery. The DUS was performed by two experienced radiologists (S.I.C., 12 years and E.J.C., 10 years for vascular DUS imaging). All imaging was performed using a HDI 5000 ultrasound (Philips Medical Systems, Bothell, WA) equipped with a high-resolution 5–9 MHz linear-array transducer, from the distal 3–4 cm of the external iliac vein to the distal calf veins. DUS included imaging in the transverse and longitudinal planes using both gray-scale and color DUS.

DVT was defined when the following conditions were seen: (a) echogenic material within lumen, (b) non-compressibility of the affected vein, or (c) nonvisualized flow in color Doppler imaging [15], [16]. To check for non-compressibility, the deep veins were evaluated at 1-cm intervals from the common femoral vein to the calf veins. At times, blood flow echogenicity resulting from blood stasis and erythrocyte aggregation contributed to false-positive results. In these conditions, dynamic tests such as flow augmentation produced by passive limb raising or upstream muscle compression were performed to exclude false positive results [17].

A routine postoperative follow-up visit was performed at 4 weeks (window period, ± 1 week) following surgery and then every 3–6 months. Symptoms and signs related to VTE were checked during the surgical admission and outpatient clinic follow-up periods. Whenever VTE-related symptoms were clinically suspected, the study protocol guided that a DUS or computed tomography angiography (CT angiography) for low extremities or pulmonary vasculature should be conducted.

### Patient population; a retrospective cohort (cohort B)

**Figure 1:**
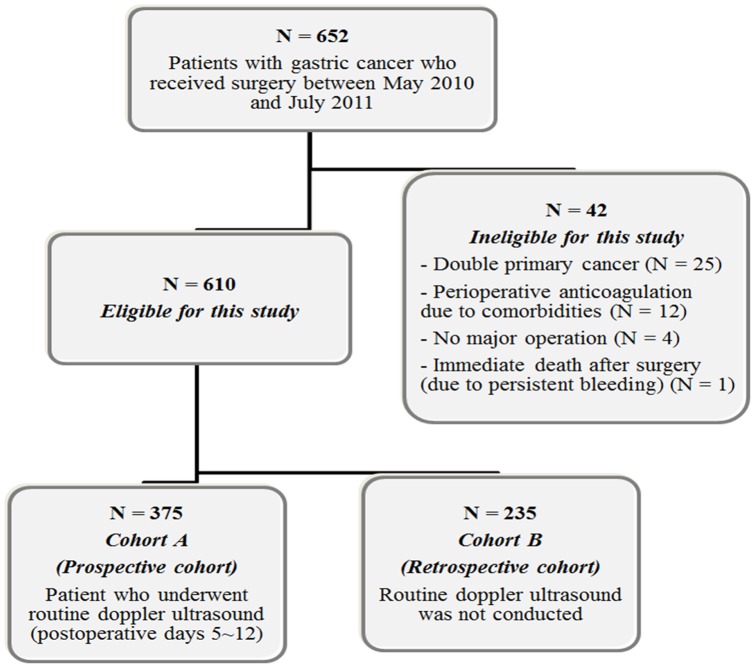
Flow of patients in this study.

During the study period, 235 patients who did not want to undergo DUS following surgery were registered to cohort B (Figure 1). These patients met the same eligibility criteria as patients in cohort A. Data collection in cohort B patients was done to reaffirm the result observed in cohort A. In cohort B, DUS or CT angiography was only performed for patients with suspected symptoms related to VTE. Most of the clinical data for cohort B were retrieved from the prospectively maintained database in the department of surgery at SNUBH [8]. However, data on VTE development were retrospectively collected from an electronic medical chart review.

### Statistical and ethical considerations

The primary objective was to find the incidence of symptomatic or asymptomatic VTE following surgery in cohort A patients. The incidence of postoperative VTE was defined as the cases detected by routine DUS (on days 5–12 after surgery) plus any additional VTE cases detected by DUS or other studies (until 4 weeks after surgery). The secondary objective was to identify risk factors for the development of VTE in this population.

We assumed that the actual incidence of postoperative VTE would be approximately 6% and this incidence would be lower than 10%. The calculated sample size was 375 with 80% power and one-sided significance level of 0.025. Although the enrollment of 420 patients was initially planned in consideration for a 10% drop-out rate, this study was completed when 375 cohort A patients were enrolled because there had been no drop-out cases at 4 weeks (window period, ± 1 week) after surgery.

Chi-square or linear-by-linear association tests were conducted to compare percentages in cross-tabulations and the t-test was used to compare means. In a multivariate analysis to investigate risk factors for VTE, a logistic regression model was applied. Two-sided *P*-values ≤ 0.05 were considered significant and SPSS software was used (SPSS, Inc., Chicago, IL). The declaration of Helsinki was followed for this study. The patients in cohort A provided written informed consent prior to this study. The exemption of acquiring written consents from cohort B patients was permitted by the Institutional Review Board (IRB) at SNUBH (IRB study number: B-1002/094-007). This study was approved by IRB and was registered to ClinicalTrials.gov (NCT01345773).

## Results

### Patient characteristics and incidence of VTE in the cohort A

Among the 610 patients who met eligibility criteria, 375 were enrolled in cohort A (Figure 1). Patient characteristics are presented in Table 1. In the cohort A, stage distribution was as follows: stage I (58.4%); stage II (15.5%); stage III (17.9%); stage IV (8.3%). Laparoscopic surgery was performed in 74.4%. A partial gastrectomy (subtotal or proximal gastrectomy) was conducted in 75.2%. The mean operation time was 179 minutes. There was no postoperative mortality. All patients (N = 375) were followed up at 4 weeks (window period, ± 1 week) and 366 patients (97.6%) were followed up at 12 weeks after surgery. Of the 9 patients whose follow-up was lost at 3 months, 6 patients were transferred to hospitals near the patients' residence and 3 patients died from cancer progression.

**Table 1:** Patient characteristics.

Variable	Cohort A (N = 375)	Cohort B (N = 235)	*P*-value
Gender			0.874*
Male	253 (67.5%)	160 (68.1%)	
Female	122 (32.5%)	75 (31.9%)	
Age			0.995*
Median (range)	61 (23–88)	62 (22–91)	
< 70 years	268 (71.5%)	168 (71.5%)	
≥ 70 years	107 (28.5%)	67 (28.5%)	
No. of comorbidites			0.779*
0	117 (31.2%)	66 (28.1%)	
1	121 (32.3%)	79 (33.6%)	
2	84 (22.4%)	59 (25.1%)	
≥ 3	53 (14.1%)	31 (13.2%)	
Preoperative laboratory [mean ± (SD)]			
White blood cell (/μL)	6832 (± 2227)	6782 (± 2129)	0.781^†^
Hemoglobin (g/dL)	13.5 (± 2.1)	13.4 (± 2.3)	0.514^†^
Platelet (× 10^3^/μL)	240 (± 82)	250 (± 77)	0.145^†^
Stage			0.324*
Stage I	219 (58.4%)	150 (63.8%)	
Stage II	58 (15.5%)	26 (11.1%)	
Stage III	67 (17.9%)	44 (18.7%)	
Stage IV	31 (8.3%)	15 (6.4%)	
Tumor location in stomach			0.257*
Low third	211 (56.3%)	139 (59.1%)	
Middle third	84 (22.4%)	58 (24.7%)	
Upper third	72 (19.2%)	31 (13.2%)	
Whole	8 (2.1%)	7 (3.0%)	
Gross morphology			0.094*
Early gastric cancer	196 (52.3%)	139 (59.1%)	
Borrmann type 1	12 (3.2%)	2 (0.9%)	
Borrmann type 2	23 (6.1%)	16 (6.8%)	
Borrmann type 3	121 (32.3%)	71 (30.2%)	
Borrmann type 4	23 (6.1%)	7 (3.0%)	
Lauren classification			0.946*
Intestinal	181 (48.3%)	113 (48.1%)	
Diffuse	176 (46.9%)	112 (47.7%)	
Mixed	18 (4.8%)	10 (4.3%)	
Tumor differentiation			0.054*
Well differentiated	41 (10.9%)	33 (14.0%)	
Moderately differentiated	138 (36.8%)	81 (34.5%)	
Poorly differentiated	131 (34.9%)	74 (31.5%)	
Signet ring cell	51 (13.6%)	45 (19.1%)	
Mucinous or other	14 (3.7%)	2 (0.9%)	
Surgical extent			0.021*
Partial gastrectomy	282 (75.2%)	197 (83.8%)	
Total gastrectomy	87 (23.2%)	33 (14.0%)	
Other palliative surgery (no gastrectomy)	6 (1.6%)	5 (2.1%)	
Surgical procedure			0.026*
Laparoscopic surgery	279 (74.4%)	193 (82.1%)	
Open surgery	96 (25.6%)	42 (17.9%)	
Operation duration			
Mean (± SD, minutes)	179 ± 60	175 ± 58	0.495^†^
≤ 2 hours	80 (21.3%)	48 (20.4%)	0.180*
> 2 and ≤ 3 hours	142 (37.9%)	106 (45.1%)	
> 3 hours	153 (40.8%)	81 (34.5%)	
Surgical outcome			0.636*
R0	349 (93.1%)	221 (94%)	
R1/R2	26 (6.9%)	14 (6.0%)	

Abbreviations: SD, standard deviation.

* chi-square test, ^†^t-test.

In multivariate analysis using a logistic regression model, the clinical parameters with P<0.20 in univariate analyses (age, number of comorbidities, WBC counts, hemoglobin level, surgical procedure [laparoscopic vs. open surgery] and stage) were included. A backward stepwise conditional logistic regression was used with P = 0.10 as the entry and P = 0.10 as the removal criteria.

Abbreviations: OR, odds ratio; CI, confidence interval; WBC, white blood cell.

In cohort A, VTE was detected in 9 patients [2.4%; 95% confidence interval (CI): 0.9–3.9] within the 4 weeks after surgery. The median time to VTE detection was 7 days (range, 6–25). VTE was detected in 8 patients by routine DUS; all 8 DVT events were asymptomatic distal calf vein thrombosis, but one patient had subtle dyspnea and a CT revealed a PE simultaneously. In the remaining one patient, on the 25^th^ day after surgery, asymptomatic PE was incidentally detected in a chest CT performed to evaluate the tumor status before initiating palliative chemotherapy and an asymptomatic proximal DVT was also simultaneously detected by additional DUS. No other VTE case was detected between 4 and 12 weeks after surgery. The further detailed characteristics of VTE events are shown in Table 2.

**Table 2:** Characteristics of patients who developed VTE during postoperative periods.

Group	Sex/Age	Comorbidity	Stage	Surgery	Surgical outcome	Operation time	Time to ambulation	VTE event	Time to VTE events after surgery	Treatment
Cohort A	F/48	Hypertension	pT1aN0M0, Stage IA	Laparoscopic subtotal gastrectomy	R0	120 min	Within 1 day	Asymptomatic DVT, - Left calf vein	7 days	Observation, spontaneously disappeared
Cohort A	F/73	Parkinson's disease	pT1aN0M0, Stage IA	Laparoscopic subtotal gastrectomy	R0	155 min	Within 1 day	Asymptomatic DVT, - Left calf vein	7 days	Observation, spontaneously disappeared
Cohort A	M/43	None	pT2N0M0, Stage IB	Laparoscopic subtotal gastrectomy	R0	160 min	Within 1 day	Asymptomatic DVT, - Right calf vein	7 days	Observation, spontaneously disappeared
Cohort A	M/73	Diabetes, Hypertension, Anemia	pT3N2M0, Stage IIIA	Laparoscopic total gastrectomy	R0	360 min	Within 1 day	Asymptomatic DVT, - Right calf vein	6 days	LMWH, disappeared
Cohort A	F/76	Anemia	pT4aN3bM0, Stage IIIC	Open total gastrectomy	R0	170 min	Within 1 day	Asymptomatic DVT, - Both calf veins	7 days	LMWH, disappeared
Cohort A	M/66	Hypertension, Anemia, Peripheral arterial disease	pT4aN3bM0, Stage IIIC	Open total gastrectomy	R0	325 min	Within 2 days	Asymptomatic DVT, - Right calf vein	7 days	LMWH, disappeared
Cohort A	M/53	Obesity, Anemia	pT4aN3bM1, Stage IV	Open total gastrectomy	R2	165 min	Within 2 days	Asymptomatic DVT, - Left calf vein	7 days	LMWH, disappeared
Cohort A	F/76	Hypertension, Congestive heart failure, Atrial fibrillation Anemia	pT4aN2M1, Stage IV	Open total gastrectomy	R2	165 min	Within 1 day	Asymptomatic DVT,- Right trifurcation level and calf veins Symptomatic PE	6 days	LMWH, disappeared
Cohort A	M/78	Hypertension, Anemia	cT4N+M1, Stage IV	Laparoscopic gastrojejunostomy	R2	55 min	Within 1 day	Asymptomatic DVT,- Left superficial femoral vein to tibioperoneal trunk and peroneal vein Asymptomatic PE	25 days	LMWH, disappeared
Cohort B	M/68	None	pT4bN2M0, Stage IIIB	Open total gastrectomy	R0	340 min	Within 3 days	Asymptomatic PE	4 days	Observation, spontaneously disappeared

Abbreviations: VTE, venous thromboembolism; DVT, deep vein thrombosis; PE, pulmonary embolism; LMWH, low molecular weight heparin.

### Risk factors for VTE development

Risk factors for postoperative VTE were analyzed in cohort A patients. The results of univariate analyses are presented in Table 3. The tumor stage was a significant factor related to VTE development (*P* = 0.008). Compared with the low VTE incidences in patients with stage I (1.4%) and II/III (2.4%), stage IV patients had 9.7% of VTE incidence. The number of comorbidities showed a borderline significance for developing VTE (*P* = 0.086). Patients aged ≥ 70 years had higher incidences of postoperative VTE than those aged < 70 years (4.7% vs. 1.5%); however, this was not statistically significant (*P* = 0.126). Laparoscopic surgery showed a numerically lower VTE incidence than open surgery, but this was not also significant (*P* = 0.190). Although the surgical outcomes (R0 vs. R1/R2) and extent (partial gastrectomy, total gastrectomy or palliative surgery without a gastrectomy) were associated with different postoperative VTE incidences (*P*<0.05), those parameters were clearly correlated with tumor stages. All patients who received palliative surgery without a gastrectomy (N = 6) and the 22 of 26 patients who had postoperative residual tumors (R1/R2 surgery) had stage IV. When 344 patients with stage I to III were separately analyzed, the extent of the surgery did not influence the postoperative VTE incidence [total gastrectomy (3/70, 4.3%) vs. partial gastrectomy (3/274, 1.1%); *P* = 0.101].

**Table 3:** The incidence of VTE according to clinical parameters.

Variable	VTE (-) (N = 366)	VTE (+) (N = 9)		*P*-value
Gender				0.480*
Male	248 (98.0%)	5 (2.0%)		
Female	118 (96.7%)	4 (3.3%)		
Age				0.126*
< 70 years	264 (98.5%)	4 (1.5%)		
≥ 70 years	102 (95.3%)	5 (4.7%)		
BMI				0.236^†^
BMI < 21	85 (96.6%)	3 (3.4%)		
21≤ BMI < 25	171 (97.2%)	5 (2.8%)		
BMI ≥ 25	110 (99.1%)	1 (0.9%)		
Smoking				0.893^†^
Never smoker	228 (97.4%)	6 (2.6%)		
Ex-smoker	59 (98.3%)	1 (1.7%)		
Current smoker	79 (97.5%)	2 (2.5%)		
No. of comorbidities				0.086^†^
0	116 (99.1%)	1 (0.9%)		
1	118 (97.5%)	3 (2.5%)		
2	82 (97.6%)	2 (2.4%)		
≥ 3	50 (94.3%)	3 (5.7%)		
WBC counts (/μL)				0.188^†^
4000 ≤ WBC < 10000	329 (97.9%)	7 (2.1%)		
WBC < 4000	13 (92.9%)	1 (7.1%)		
WBC ≥ 10000	24 (96.0%)	1 (4.0%)		
Hemoglobin (g/dL)				0.140*
Hemoglobin ≥ 10.0	340 (98.0%)	7 (2.0%)		
Hemoglobin < 10.0	26 (92.9%)	2 (7.1%)		
Platelet counts (× 10^3^/μL)				0.957^†^
130 ≤ Platelet < 400	341 (97.7%)	8 (2.3%)		
Platelet < 130	13 (100.0%)	0 (0.0%)		
Platelet ≥ 400	12 (92.3%)	1 (7.7%)		
Stage				0.008^†^
Stage I	216 (98.6%)	3 (1.4%)		
Stage II/III	122 (97.6%)	3 (2.4%)		
Stage IV	28 (90.3%)	3 (9.7%)		
Tumor location in stomach				0.278^†^
Low third	217 (98.6%)	3 (1.4%)		
Middle third	61 (95.3%)	3 (4.7%)		
Upper third	79 (96.3%)	3 (3.7%)		
Whole	9 (100.0%)	0 (0.0%)		
Gross morphology				0.271^†^
Early gastric cancer	193 (98.5%)	3 (1.5%)		
Borrmann type 1	11 (91.7%)	1 (8.3%)		
Borrmann type 2	23 (100.0%)	0 (0.0%)		
Borrmann type 3	117 (96.7%)	4 (3.3%)		
Borrmann type 4	22 (95.7%)	1 (4.3%)		
Lauren classification				0.531^†^
Intestinal	176 (97.2%)	5 (2.8%)		
Diffuse	172 (97.7%)	4 (2.3%)		
Mixed	18 (100%)	0 (0%)		
Tumor differentiation				0.632^†^
Well differentiated	41 (100%)	0 (0%)		
Moderately differentiated	133 (96.4%)	5 (3.6%)		
Poorly differentiated	129 (98.5%)	2 (1.5%)		
Signet ring cell	50 (98.0%)	1 (2.0%)		
Mucinous or other	13 (92.9%)	1 (7.1%)		
Surgical extent				0.001^†^
Partial gastrectomy‡	279 (98.9%)	3 (1.1%)		
Total gastrectomy	82 (94.3%)	5 (5.7%)		
Other palliative surgery (no gastrectomy)	5 (83.3%)	1 (16.7%)		
Surgical procedure				0.190*
Laparoscopic surgery	274 (98.2%)		5 (1.8%)	
Open surgery	92 (95.8%)		4 (4.2%)	
Operation duration				0.440^†^
≤ 2 hours	78 (97.5%)		2 (2.5%)	
> 2 and ≤ 3 hours	137 (96.5%)		5 (3.5%)	
> 3 hours	151 (98.7%)		2 (1.3%)	
Surgical outcome				0.019*
R0	343 (98.3%)		6 (1.7%)	
R1/R2	23 (88.5%)		3 (11.5%)	
Time to ambulation after surgery				0.682^†^
≤ 24 hours	271 (97.5%)		7 (2.5%)	
> 24 and ≤ 48 hours	82 (97.6%)		2 (2.4%)	
> 72 hours	13 (100%)		0 (0%)	

* Fisher's exact test, ^†^linear-by-linear association.

‡ Subtotal gastrectomy was conducted in 271 patients and proximal gastrectomy in 11 patients.

Abbreviations: VTE, venous thromboembolism; BMI, body mass index; WBC, white bleed cell.

In multivariate analysis, the clinical parameters with *P*<0.20 in univariate analyses were included (Table 4). Only the disease stage was predictive of postoperative VTE development: patients with stage IV had a higher incidence of postoperative VTE with an odds ratio (OR) of 8.18 (95% CI, 1.54–43.42) compared with those with stage I. However, the risk of VTE in patients with stage II/III was not different from those with stage I. Although there was no statistical significance, elderly patients (age ≥ 70 years) had a trend of developing higher VTE than patients aged < 70 years (OR 3.42; 95% CI, 0.88–13.33; *P* = 0.076).

**Table 4:** Multivariate analysis (logistic regression analysis) for the postoperative development of venous thromboembolism.

	OR	95% CI	*P*-value
Age			
< 70 years	1.00	–	–
≥ 70 years	3.42	0.88–13.33	0.076
Stage			
stage I	1.00	–	–
stage II/III	1.68	0.33–8.53	0.529
stage IV	8.18	1.54–43.42	0.014
Constant	0.008	–	< 0.001

### The development of VTE in the cohort B

Compared with patients in the cohort A, the extent of gastrectomy and surgical procedure (laparoscopic vs. open surgery) showed a different distribution in cohort B patients (N = 235, Table 1). At 4 weeks, the dropout rate during follow-up was 1.3% (3/235). Three patients dropped out before the 21^st^ day after surgery without suspected VTE symptoms or postoperative complications; these 3 patients were referred to nearby hospitals for the further follow-up.

In the cohort B, symptomatic postoperative VTE did not develop. Only one case of asymptomatic PE in a segmental branch of the right lower lobe pulmonary artery was incidentally found in an abdominal CT performed to evaluate postoperative complications. The PE in this case was spontaneously resolved without treatment (Table 2).

## Discussion

This is the largest prospective study on the incidence of postoperative VTE in GC patients. It demonstrated that postoperative VTE is very rare (2.4%; 95% CI, 0.9–3.9) in Korean GC patients. As routine pharmacologic prophylaxis is generally considered when the incidence of postoperative VTE is ≥ 10% [10], our study shows that risk-stratified applications of perioperative pharmacologic thromboprophylaxis is more appropriate than the routine pharmacologic thromboprophylaxis in Asian GC patients receiving surgery.

It has been demonstrated that Asians have a lower incidence of VTE [3], [8], [11], [12], [13], [18], [19], [20], [21], [22], [23], [24]. In a Korean prospective study which included 107 patients with various gastrointestinal cancers, the postoperative VTE incidence detected by DUS was 7.5% [21]. Our previous retrospective studies on patients with stomach or colorectal cancer reported the incidence of postoperative VTE much lower than Western patients [8], [11]. Another Korean study by Jeong et al. reported no cases of symptomatic VTE among 182 GC patients following a gastrectomy that had not received LMWH prophylaxis [24]. In the present study, the incidence of postoperative VTE detected by DUS was only 2.4% in prospective cohort A (N = 375). To reaffirm the low incidence of VTE observed in cohort A, a separate analysis on retrospective cohort B (N = 235) was done and only one patient (0.4%) was found to have postoperative VTE. Our study clearly shows that the incidence of postoperative VTE in Korean GC patients is much lower than that of Western patients.

In our study, only the disease stage was predictive of postoperative VTE and the incidence of VTE tended to increase in elderly patients in the multivariate analysis (Table 4). An advanced stage has been consistently reported to be predictive of VTE in previous studies [3], [5], [8], [11], [12], [13], [22] and an older age is also a well-known risk factor [3], [5], [8], [12], [13], [19], [25], [26]. Considering the low incidence of overall postoperative VTE in our patient cohorts, the risk-stratified application of perioperative pharmacologic thromboprophylaxis for selected GC patients such as those with stage IV are thought to be more appropriate in Korea than routine pharmacologic thromboprophylaxis.

Although GC has been reported to have high risk of VTE development in Western studies [27], [28], the reasons why the incidence of postoperative VTE in our Korean GC patients is too low need to be further discussed. In a Japanese study conducted on abdominal surgery patients that consisted of general, gynecologic and urologic surgery (N = 173), the VTE incidence detected by venography was 24.3%, which was almost comparable to ranges reported in the West [29]. Use of a venography, having a higher sensitivity for VTE detection than DUS, may be one of the reasons for an increased VTE detection as compared to our study. However, DUS is the current standard method for VTE detection as a venography is a cumbersome procedure. Including many intra-pelvic surgery cases (53%), which was related to more frequent postoperative VTE development than upper abdominal surgery, may be another reason of increased detection of VTE. Moreover, the number of GC patients recruited in that study was too small (N = 33) [29]. Therefore, results of the Japanese study cannot be generalized to Asian GC patients. The lower incidence of postoperative VTE in our study might be attributed to the application of mechanical thromboprophylaxis (elastic bandage or stockings), tumor characteristics and a frequent use of laparoscopic surgery. The Japanese study had mechanical thromboprophylaxis performed in about half of the patients [29], whereas mechanical prophylaxis was routinely used for our study. Although there are conflicting results, these mechanical methods might have played an important role in reducing postoperative VTE in our study [24], [30], [31]. Mechanical thromboprophylaxis has been preferred to pharmacologic thromboprophylaxis by most Asian surgeons because of concerns about increased postoperative bleeding related to LMWH [20], [24], [31]. Another reason for the low incidence of postoperative VTE in Korean GC patients may be due to the increased number of cases with early GC (EGC). Since endoscopic surveillance is commonly conducted for early diagnosis of GC in Asian countries including Korea and Japan [32], EGC becomes more common. In the present study, stage I disease was about 60% and this proportion of EGC is in a similar range to those reported from other Korean institutions [33], [34], [35], [36]. Japan is known to have a higher proportion of EGC cases as compared to Korea [37], [38]. As advanced stage is most predictive of VTE development in GC [8], [22], the high prevalence of EGC in eastern Asian countries may be one of reasons for decreased postoperative VTE. In addition, a frequent use of laparoscopic gastrectomy in the patient population might be another explanation. In retrospective studies mostly composed of patients with benign diseases, a lower incidence of VTE after laparoscopic surgery compared with open surgery was reported [39], [40]. In Asian countries including Korea and Japan, despite the lack of long term survival data from well-designed randomized trials [41], [42], [43], a laparoscopy-assisted gastrectomy for EGC is rapidly gaining popularity based on the benefits of a shorter hospital stay, earlier mobilization and functional recovery. As a large-scaled phase 3 study comparing laparoscopic and open surgery in EGC patients has completed patient enrollment and is awaiting survival outcomes [43], laparoscopic gastrectomy is expected to be more popular if long term survival outcomes are shown to be similar between laparoscopic and open gastrectomy. In the present study, 74.4% of patients received laparoscopic surgery and 74.1% of patients were able to ambulate within 24 hours after surgery (Table 3).

Although our study was conducted at a single institution, the situations at other Korean institutions are similar to our institution as most Korean GC patients receive surgery at experienced tertiary high-volume centers [44]. Therefore, our results are thought to be generalized in Korea [21], [24]. However, the generalization of our results for all Asian cancer patients needs to be very cautious. In large prospective studies conducted for Asian patients receiving major orthopedic surgery, postoperative VTE incidence was in a range similar to that of Western patients; the aggressiveness of orthopedic surgery and prolonged immobilization are thought to overwhelm the ethnic advantage of Asian patients [45], [46]. As mentioned above, intra-pelvic surgery was reported to be related to a higher postoperative VTE than upper abdominal surgery [29]. In addition, the propensity of developing VTE may be different according to different ethnic groups even within Asian countries [46]. Therefore, prospective studies on the necessity of routine perioperative pharmacologic thromboprophylaxis in Asian cancer patients need to be conducted, probably in each Asian country separately.

In summary, the incidence of postoperative VTE was 2.4% in Korean GC patients and only advanced stage was related to the frequent development of postoperative VTE. Risk-stratified applications of perioperative pharmacologic thromboprophylaxis are thought to be appropriate in Korean GC patients. More prospective studies on the postoperative incidence of VTE in Asian cancer patients are also warranted.
